# The Germinal Center Kinase GCK-1 Is a Negative Regulator of MAP Kinase Activation and Apoptosis in the *C. elegans* Germline

**DOI:** 10.1371/journal.pone.0007450

**Published:** 2009-10-14

**Authors:** Katherine R. Schouest, Yasuhiro Kurasawa, Tokiko Furuta, Naoki Hisamoto, Kunihiro Matsumoto, Jill M. Schumacher

**Affiliations:** 1 Department of Genetics, The University of M.D. Anderson Cancer Center, Houston, Texas, United States of America; 2 Genes and Development Program, University of Texas Graduate School of Biomedical Sciences at Houston, Houston, Texas, United States of America; 3 Department of Molecular Biology, Graduate School of Science, Institute for Advanced Research, Nagoya University, Nagoya, Japan; 4 CREST, Japan Science and Technology Corporation, Chikusa-ku, Nagoya, Japan; University of Missouri-Kansas City, United States of America

## Abstract

The germinal center kinases (GCK) constitute a large, highly conserved family of proteins that has been implicated in a wide variety of cellular processes including cell growth and proliferation, polarity, migration, and stress responses. Although diverse, these functions have been attributed to an evolutionarily conserved role for GCKs in the activation of ERK, JNK, and p38 MAP kinase pathways. In addition, multiple GCKs from different species promote apoptotic cell death. In contrast to these paradigms, we found that a *C. elegans* GCK, GCK-1, functions to inhibit MAP kinase activation and apoptosis in the *C. elegans* germline. In the absence of GCK-1, a specific MAP kinase isoform is ectopically activated and oocytes undergo abnormal development. Moreover, GCK-1- deficient animals display a significant increase in germ cell death. Our results suggest that individual germinal center kinases act in mechanistically distinct ways and that these functions are likely to depend on organ- and developmental-specific contexts.

## Introduction

The Ste20-related germinal center kinases comprise a large protein family that has been implicated in cellular processes ranging from cytoskeletal dynamics and stress responses, to cell growth, proliferation, and death [1]–[4]. The founding member, *S. cerevisiae* Ste20, activates MAP kinase signaling in response to mating pheromone as a MAP kinase kinase kinase kinase (MAP4K) upstream of the MAP3K Ste11p [5], [6]. Dozens of proteins with kinase domains highly similar to Ste20 were subsequently found in invertebrates and vertebrates [1]. Based on the location of this conserved kinase domain and the presence or absence of a p21-activated kinase (PAK) domain, these proteins have been divided into two large families, PAKs and germinal center kinases (GCKs) [7]. Based on slightly different kinase signature sequences and divergence outside the conserved kinase domain, the GCKs have been further grouped into eight distinct subfamilies (GCK I – GCK VIII) that (with the exception of subfamily VII) consist of one *C. elegans*, one *Drosophila*, and multiple vertebrate/mammalian proteins [7].

Like the founding kinase Ste20, members of the GCK I, II, III, IV, V, VII, and VIII subfamilies have been reported to activate MAP kinase signaling, including JNK, p38, and ERK pathways [7]–[15]. Likewise, members of multiple subfamilies are potent inducers of apoptosis [2], [7], [16], [17]. However, there are exceptions to these observations. Although two human members of the GCK VII family, TAO1 and TAO2 activate p38 [14], [15], a third human GCK VII kinase, JIK, appears to inhibit JNK signaling [18]. Interestingly, while the human GCK V kinase SLK induces apoptosis when overexpressed [17], expression of the highly related Drosophila SLIK kinase inhibits cell death [19]. Though some of these conflicting results could be due to species-specific differences, caveats remain since the majority of the existing data has been gleaned from over-expression studies in tissue culture coupled with in vitro kinase assays [2], [7]. Clearly, addressing the physiological role of these kinases in the context of a whole organism will be essential for parsing the functions of each family member.

In addition to intracellular events, MAP kinase signaling pathways regulate a remarkable number of developmental processes [20], [21]. For instance, a conserved ERK signaling cascade is essential for oocyte maturation in both vertebrates and invertebrates [22], [23]. In *C. elegans,* ERK is activated at two distinct times during oocyte development: first, as germ cells progress through pachytene, and second, in maturing diakinetic oocytes residing proximal to the spermatheca [24], [25]. Mutation or depletion via RNA mediated interference (RNAi) of the *C. elegans* ERK ortholog MPK-1 and other components of the ERK cascade results in sterility characterized by the failure of germ cells to progress through pachytene [24], [26]. The affected nuclei clump together and eventually disintegrate [26]. MAP kinase activation in pachytene is also required for a developmentally programmed germ cell death switch that reduces the number of maturing oocytes by one half [27].

It has been recognized for a number of years that *C. elegans* oocyte maturation is regulated by sperm [28]. Recently, elegant biochemical analyses yielded the surprising result that the Major Sperm Protein (MSP), a highly abundant, sperm-specific cytoskeletal protein, is released from intact sperm and is required for MAP kinase activation, oocyte maturation, and ovulation [25], [29]. In the absence of MSP, ephrin binding of VAB-1, an Eph receptor protein-tyrosine kinase, and a parallel pathway regulated by CEH-18, a POU-class homeoprotein, inhibit MAP kinase signaling [30]. MSP binding relieves VAB-1 inhibition, leading to MAP kinase activation [30]. Together, these inhibitory pathways ensure that MAP kinase activation and oocyte maturation are tightly linked to the presence of sperm. The upstream signal required for MAP kinase activation during pachytene is not known.

Here we report that MAP kinase activation in pachytene is inhibited by GCK-1, the sole *C. elegans* member of the GCK III subfamily. Loss of GCK-1 results in the hyper-activation of a specific MAP ERK kinase isoform, abnormal oocyte development, and increased germ cell death. Altogether, this study has uncovered a novel role for a germinal center kinase in germ cell development and reveals that GCK kinases can have opposing roles in the regulation of MAP kinase activation and apoptosis.

## Results

### T19A5.2 encodes a germinal center kinase required for oogenesis

The *C. elegans* genome encodes 8 recognizable members of the germinal center kinase family [7], [31]. One these, T19A5.2/GCK-1, can be grouped with the GCK III subfamily (Figure S1) [7]. To determine the functional role of GCK-1 during *C. elegans* development, dsRNA corresponding to the full-length *gck-1a* isoform was microinjected or fed to wild-type (wt) young adult or L4 larval stage hermaphrodites. *gck-1(RNAi)* by either method resulted in progressive sterility. The animals were completely sterile within 24 hours of RNAi induction. *gck-1(RNAi)* hermaphrodites produced broods of <70 progeny as compared to >250 progeny from untreated wt age-matched controls. The F1 progeny of *gck-1(RNAi)* animals were completely sterile, and displayed a phenotype that was indistinguishable from their sterile mothers (below and data not shown). Consistent with these results, hermaphrodites homozygous for a *gck-1* deletion allele that lacks a significant portion of the kinase domain (*gck-1(km15)*) (Figure S1) had a similar sterile phenotype. Since animals depleted of GCK-1 activity by any of the above means display a similar phenotype (described in detail below), they will be collectively referred to as *gck-1(lf)*, except as noted.

The *C. elegans* hermaphrodite gonad consists of two U-shaped arms that share a common uterus (Figure S2). Beginning in the fourth larval stage (L4), developing germ cells within the gonad undergo spermatogenesis, while at the L4 to adult molt, a genetic switch occurs and the germ cells develop as oocytes [32]. The region of the gonad most distal to the uterus contains proliferating germ nuclei which then transition into meiosis with crescent shapes characteristic of the leptotene and zygotene stages of meiotic prophase I [33]. As the nuclei pass through the gonad, they enter into pachytene, where pairing of thread-like chromosomes becomes evident. Pachytene nuclei are arranged in an orderly fashion on the gonad surface, forming a tube surrounding a common anucleate cytoplasm, the rachis [34]. The rachis terminates at the exit from pachytene, usually near the bend in the gonad arm, although small connections between the oocytes and the syncytial cytoplasm remain [35]. Upon pachytene exit, approximately 50% of the developing oocytes undergo apoptosis [27]. The surviving nuclei become cellularized and organized into a single row as they grow and progress through diplotene into diakinesis. These oocytes remain in diapause with six highly condensed bivalent chromosomes until a maturation signal is received from sperm [25], [28].

**Figure 1:**
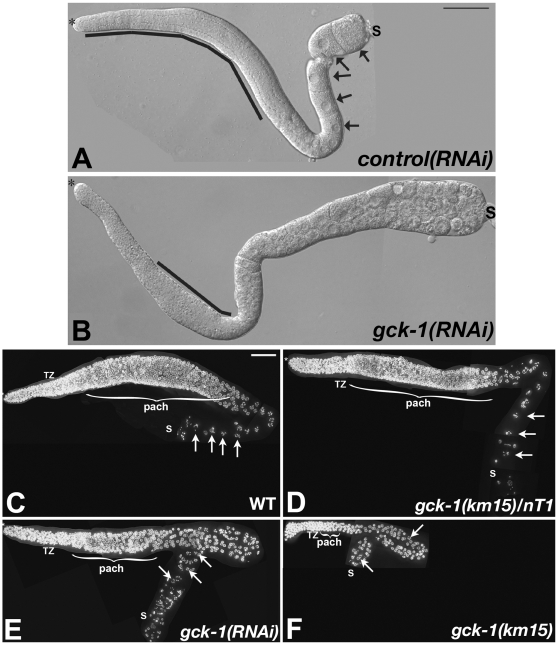
*gck-1(lf)* hermaphrodites are sterile. (A) Wt and (B) *gck-1(RNAi)* dissected gonads visualized by DIC optics. *, distal tip; line, rachis; arrows, growing oocytes; S, spermatheca. (C) Wt, (D) *gck-1(km15)/*nT1, (E) *gck-1(RNAi)*, and (F) *gck-1(km15)* dissected gonads stained with DAPI. Deconvolved, flattened image stacks are shown. *, distal tip; TZ, transition zone; }pach, pachytene; arrows, diakinetic or diakinetic-like nuclei; S, spermatheca. Scale bars, 20 µm.

To determine the cause of sterility in *gck-1(lf)* animals, gonads dissected from wt and *gck-1(RNAi)* hermaphrodites were examined by differential interference contrast (DIC) microscopy (Figure 1A,B). Each wt gonad displayed a highly ordered progression of germ cells through meiotic prophase with a clearly visible rachis and a single row of growing oocytes in the proximal gonad (Figure 1A, arrows). In contrast, gonads from *gck-1(lf)* animals had little to no discernable rachis, and the proximal ends were filled with dozens of small germ cells (Figure 1B).

To characterize *gck-1(lf)* germline defects, gonads dissected from wt, *gck-1(RNAi), gck-1(km15)* heterozygotes, and *gck-1(km15)* homozygous animals were fixed, stained with DAPI, and examined by deconvolution microscopy (Figure 1C–F). This analysis revealed that *gck-1(km15)*/nT1, *gck-1(RNAi)*, and *gck-1(km15)* gonads had progressively fewer germ cells than wt (Figure S3). Differences were most pronounced in pachytene, where *gck-1(km15)/nT1* heterozygotes, *gck-1(RNAi)*, and *gck-1(km15)* gonads had 35, 60, and 81% fewer pachytene nuclei respectively than wt (Supplemental Table S1). Although there was not a statistically significant difference in the number of mitotic metaphase figures in all four genotypes/conditions (Figure S3), the number of nuclei at all other stages of oogenesis was decreased in *gck-1(RNAi)* and *gck-1(km15)* gonads (Figure S3 and Table S1). *gck-1(lf)* gonads also contained a large number of nuclei whose stage in meiotic prophase could not be readily determined by morphology or position (Figures 1E,F and S3; Table S1).

Although the pachytene region of *gck-1(km15)*/nT1 heterozygotes contained fewer germ cells, all other aspects of oogenesis were grossly normal and these animals were fertile (Figure 1D). However, in *gck-1(RNAi)* and *gck-1(km15)* animals, striking morphological differences from wt became apparent during pachytene (Figure 1E,F). Unlike wt, *gck-1(lf)* nuclei with paired, thread-like chromosomes were not arranged in an orderly pattern on the gonad periphery, and there was no clear transition from pachytene into diplotene (usually just prior to the bend in wt gonad arms) (Figure 1C–F). Moreover, unlike the single row of growing oocytes found in wt and *gck-1(km15)/nT1* heterozygotes (Figure 1A,C,D, arrows), the proximal gonads of *gck-1(lf)* hermaphrodites contained a large number of small cells/oocytes (Figure 1B,E,F). While some of these cells had diakinetic-like chromosomes (Figure 1E,F, arrows), the exact stage of many of these cells could not be determined, and there was no clear progression from diplotene into diakinesis, as diakinetic-like cells were scattered throughout the proximal half of *gck-1(lf)* gonads (Figure 1E,F).

**Figure 2:**
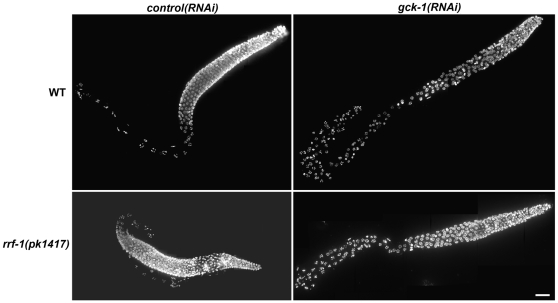
GCK-1 functions in the germline. Gonads dissected from wt and *rrf-1(pk1417)* hermaphrodites fed control or *gck-1* dsRNA were stained with DAPI. Deconvolved, flattened image stacks are shown. Scale bar, 20 µm.

To determine whether GCK-1 acts in the soma or germline to affect germ cell development, wt and *rrf-1(pk1417)* hermaphrodites were fed control and *gck-1* dsRNAs. RRF-1 encodes an RNA directed RNA polymerase that is required for RNAi in somatic cells but not the germline [36]. Hence RNAi inhibition does not affect somatically expressed genes in *rrf-1(pk1417)* mutant animals, but is still potent in the germline. DAPI staining revealed that the *gck-1(RNAi)* phenotype does not differ between wt and *rrf-1(pk1417)* gonads (Figure 2), indicating that GCK-1 functions in the germline.

**Figure 3:**
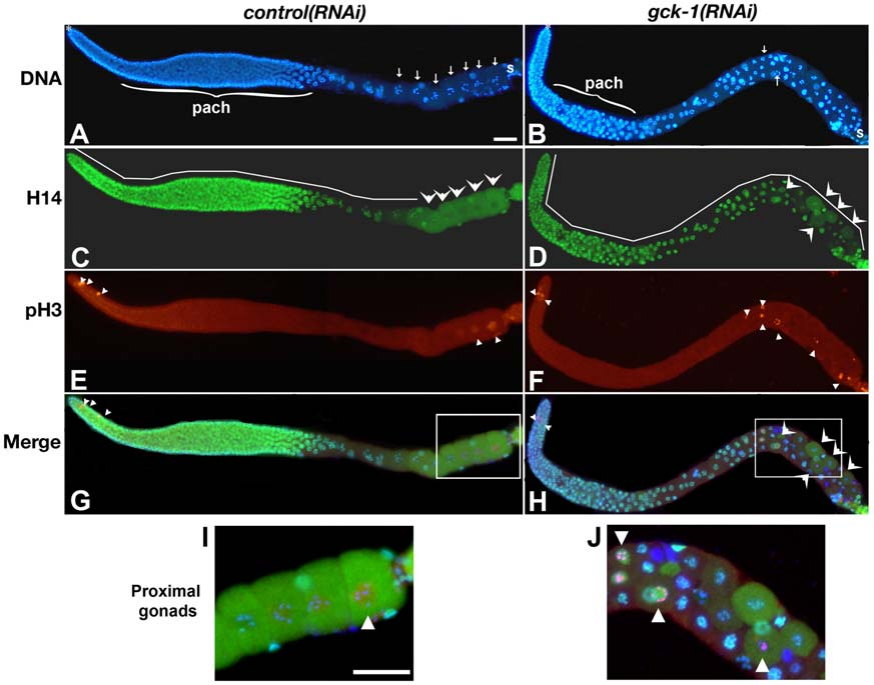
Meiotic prophase progression is disrupted in *gck-1(lf)* hermaphrodites. *Control(RNAi)* (A,C,E,G,I) and *gck-1(RNAi)* (B,D,F,H,J) dissected gonads were stained with DAPI (blue: A,B,G,H,I,J), and H14 (green: C,D,G,H,I,J), and pH 3 antibodies (red: E,F,G,H,I,J). Merged images are shown in (G,H,I,J). (I,J) Enlarged images of boxed regions in (G,H). Spermatheca is to the right in all images. *, distal tip (A,B); }pach, pachytene (A,B); line, H14 nuclear staining (C,D); large arrowheads, cytoplasmic H14 staining (C,D,H); small arrowheads, pH 3 positive cells (E,F,I,J); arrows, diakinetic and diakinetic-like nuclei (A,B); S, spermatheca; Scale bars, 20 µm.

To ascertain if the abnormal diakinetic-like oocytes in *gck-1(lf)* gonads display other characteristics of diakinetic oocytes, wt and *gck-1(RNAi)* gonads were immunostained for two markers that delineate diakinetic oocytes from earlier stages of meiotic prophase. Staining of wt hermaphrodite gonads with antibodies that recognize a phosphorylated residue on the C-terminal domain (CTD) of RNA polymerase II (H14), and histone H3 phosphorylated at serine 10 (pH 3) results in a specific pattern during *C. elegans* oogenesis [37], [38]. H14 stains germ cell chromosomes from the distal mitotic region through diplotene [37], and recognizes a cytoplasmic antigen in diakinetic oocytes. Although this cytoplasmic antigen is unlikely to be RNA polymerase, H14 staining clearly delineates diakinesis from earlier stages of meiotic prophase (Figure 3C, arrowheads). pH 3 immunostains mitotic nuclei in the distal gonad and the diakinetic chromosomes of maturing oocytes residing close to the spermatheca ([38] and Figure 3E, small arrowheads). The appearance of pH 3 on diakinetic chromosomes is sperm dependent [39]–[41]. In late diakinetic oocytes, H14 and pH 3 immunostaining patterns are complementary (Figure 3G,I). H14 staining is always cytoplasmic in oocytes with chromosomal pH 3 (Figure 3G,I).

As in wt animals, H14 immunostaining in *gck-1(lf)* gonads was associated with all germ cell nuclei from the distal mitotic region through pachytene. However, in the proximal gonad, some of the cells displayed chromosomal H14 staining while in others, it was clearly cytoplasmic (Figure 3D,H,J, arrowheads). Importantly, germ cells with cytoplasmic H14 staining nearly always had chromosomes with diakinetic-like morphology, suggesting that H14 immunostaining remains a useful marker for delineating different germ cell stages in the disorganized *gck-1(lf)* germline.

As in wt, the pH 3 antibody stained the chromosomes of *gck-1(lf)* mitotic germ cell nuclei (Figure 3E,F). However, in the *gck-1(lf)* proximal gonad, pH 3 immunostaining was not limited to oocytes residing next to the spermatheca, but was associated with the diakinetic-like chromosomes of cells scattered throughout the proximal half of *gck-1(lf)* gonads (Figure 3F, arrowheads). These scattered nuclei had the chromosome morphology and patterns of H14 and pH 3 immunostaining that are consistent with the diakinetic stage of oogenesis (Figure 3H,J).

### GCK-1 inhibits the activation of a specific MAP kinase isoform

A canonical MAP kinase pathway regulates proliferation, development, and apoptosis in the *C. elegans* germline [25], [27], [42], [43]. MAP kinase is activated by an unknown signal as germ cells transit through pachytene and again in the proximal gonad by MSP signaling in maturing oocytes [25]. Since GCK-1 affects germ cell development, the pattern and extent of MAP kinase activation was assessed in *gck-1(lf)* gonads.

**Figure 4:**
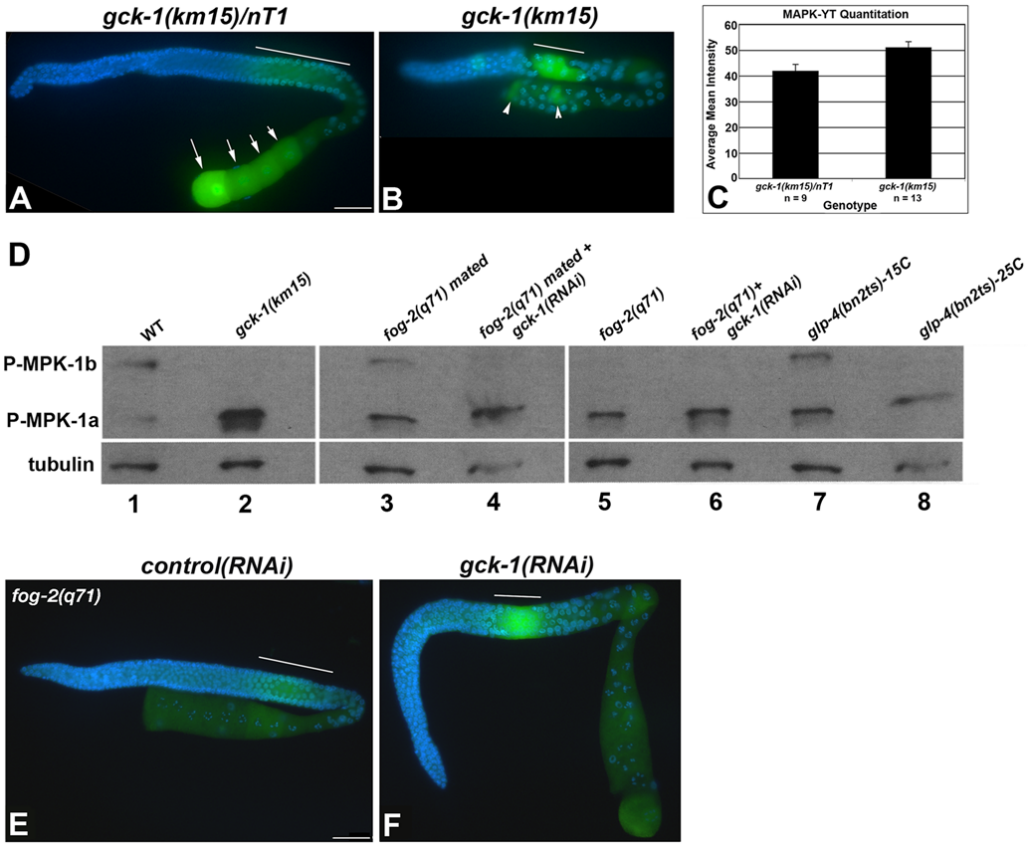
MAP kinase is hyper-activated in *gck-1(lf)* gonads. (A) *gck-1(km15)*/nT1 and (B) *gck-1(km15)* dissected gonads stained with To-Pro (blue) and the MAPK-YT antibody (green). Line, pachytene MAPK-YT immunostaining; Arrows, diakinetic oocytes (A); arrowheads, MAPK-YT positive cells (B). (C) Quantitation of pachytene MAPK-YT immunostaining intensity in *gck-1(km15);nT1* (n = 9) and *gck-1(km15)* (n = 13) gonads. (*P<0.001; error bars represent the standard error of the means.) (D) Western analysis of whole worm lysates probed with MAPK-YT and α-tubulin as a loading control. Genotypes/conditions used were (1) wt, (2) *gck-1(km15),* (3) *fog-2(q71)* mated females, (4) *gck-1(RNAi); fog-2(q71)* mated females, (5) *fog-2(q71)* females, (6) *gck-1(RNAi);fog-2(q71)* females, (7) *glp-4(bn2ts)* reared at 15°C (intact germline), and (8) *glp-4(bn2ts)* reared at 25°C (germline-less). (E,F) Gonads dissected from unmated *fog-2(q71)* females treated with control (E) and *gck-1(RNAi)* (F) stained with To-Pro (blue) and the MAPK-YT antibody (green). Line, pachytene MAPK-YT staining. Scale Bars, 20 µm.

Immunostaining of wt and *gck-1(km15)*/nT1 gonads with an antibody specific for activated MAP kinase (MAPK-YT) resulted in the expected pattern of MAP kinase activation during pachytene (Figure 4A, white line) and increasing amounts in oocytes as they progressed closer to the spermatheca, consistent with activation by a diffusible signal from sperm (Figure 4A, arrows) [25], [29]. In *gck-1(km15)* animals there was a statistically significant increase in activated MAP kinase in pachytene compared to wt (Figures 4B (white line) and 4C). In addition to pachytene, MAPK-YT immunostaining was also present in a few random germ cells in the *gck-1(km15)* proximal gonad (Figure 4B, arrowheads).

Two isoforms of *C. elegans* MAP kinase/ERK are encoded by the *mpk-1* locus (MPK-1a, 43 kD, and MPK-1b, 50 kD) and differ only by the presence of an additional 5′ exon in the larger isoform [44]. Immunostaining with the MAPK-YT antibody does not distinguish between activated MPK-1a and MPK-1b since the phosphorylated epitope is found in both isoforms. However, since the two isoforms encode proteins of different sizes, they are easily discernable by western analysis with MAPK-YT antibody (Figure 4D) [44].

Western analysis of protein extracts from wt and *gck-1(km15)* young adult hermaphrodites (L4+24 hours) revealed a significant decrease in MPK-1b activation concomitant with a striking increase in MPK-1a activation in *gck-1(km15)* animals (Figure 4D, lanes 1,2). The increased levels of MPK-1a activation in the absence of MPK-1b activation, and the increased intensity of MAPK-YT immunostaining during pachytene in *gck-1(km15)* gonads suggest that loss of GCK-1 leads to germline activation of MPK-1a specifically at pachytene. However, it was previously proposed that the MPK-1b isoform is specific for germ cell development since it is only activated in animals with a functional germline, and there were no discernible germline dependent changes in the activation of the MPK-1a isoform ([44] and Figure 4D, lanes 1,7,8). The simplest interpretation of these results is that the MPK-1a and MPK-1b isoforms are activated at distinct stages of oocyte development, with MPK-1a activation occurring in mid-pachytene by an unknown signal, while MPK-1b is activated by MSP in oocytes most proximal to the spermatheca.

To test this model, we asked whether MAPK-YT staining of maturing oocytes in the proximal gonad is due to specific activation of the MPK-1b isoform by MSP. The F-box protein FOG-2 is required for hermaphrodite spermatogenesis [45]; hence, a *fog-2(q71)* mutant strain produces fertile males and females, but no hermaphrodites [32]. Consistent with our model, western analysis revealed that MPK-1b was specifically activated in mated *fog-2(q71)* females but not unmated *fog-2(q71)* females (Figure 4D, lanes 3,5). MPK-1a activation was not affected by the presence or absence of sperm (Figure 4D, lanes 3,5).

We predicted that increased MPK-1 activation in pachytene in *gck-1(lf)* gonads should be independent of sperm and MSP signaling. To test this, MAP kinase activation was examined in unmated *fog-2(q71)* females fed *gck-1* or control dsRNA. DAPI staining confirmed that the *gck-1(RNAi);fog-2(q71)* gonads retained the *gck-1(lf)* phenotype, with multiple rows of small oocytes in the proximal gonad (Figure 4E,F). As expected MAPK-YT staining was low in the proximal gonads of both control and *gck-1(RNAi)* females, whereas there was a clear increase in pachytene MAP kinase activation in *gck-1(RNAi);fog-2(q71)* animals (Figure 4E,F). Western blotting revealed a corresponding specific increase in MPK-1a activation (Figure 4D, lane 6). A similar activation pattern was seen in mated *gck-1(RNAi);fog-2(q71)* animals (Figure 4D, lane 4, and data not shown). Altogether, these results are consistent with the hypothesis that the MPK-1a and MPK-1b isoforms are differentially activated at distinct times during oocyte development. We posit that MPK-1a activation occurs during pachytene and is inhibited by the GCK-1 kinase, while MPK-1b activation occurs in proximal oocytes and is dependent on MSP signaling.

### Oocyte development in gck-1(lf) gonads requires the MAP kinase pathway

**Figure 5:**
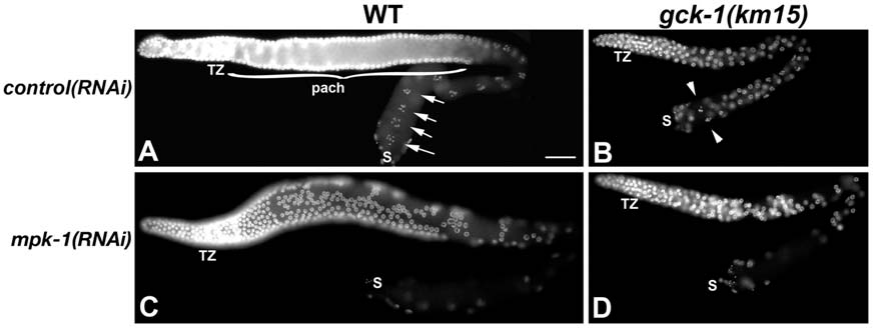
The *gck-1(lf)* phenotype requires the MAP kinase pathway. (A–D) DAPI stained gonads from (A,C) wt and (B,D) *gck-1(km15)* animals fed control (A,B) or *mpk-1* dsRNA (C,D). TZ, transition zone; bracket, pachytene; arrows, diakinetic oocytes; arrowheads, diakinetic-like nuclei; S, spermatheca. Scale Bar, 20 µm.

A second facet of our model predicts that the *gck-1(lf)* phenotype should be dependent on MAP kinase. *mpk-1* mutations and *mpk-1(RNAi)* lead to sterility characterized by defects in pachytene progression, where the “stuck” germ cell nuclei clump together and degenerate, leaving the proximal gonad devoid of nuclei [24], [26]. Hence, MPK-1 was depleted from wt and *gck-1(km15)*/nT1 heterozygous animals by feeding *mpk-1* dsRNA and their progeny were examined. All of the wt, *gck-1(km15)*/nT1, and *gck-1(km15)* hermaphrodite progeny of *mpk-1(RNAi)* treated mothers exhibited a strong pachytene arrest phenotype as evidenced by clumping of pachytene nuclei and proximal gonads devoid of germ cells (Figure 5 and data not shown). Similar results were found in *gck-1(km15)* animals treated with, Ksr/*ksr-2*, Mek/*mek-2* or Raf*/lin-45(RNAi)* (Figure S4). These results indicate that the *gck-1(lf)* phenotype is dependent on the MAP kinase signaling pathway. Hence, GCK-1 is likely to act upstream of Raf or in a parallel pathway.

### GCK-1 directly interacts with MPK-1 in vitro

**Figure 6:**
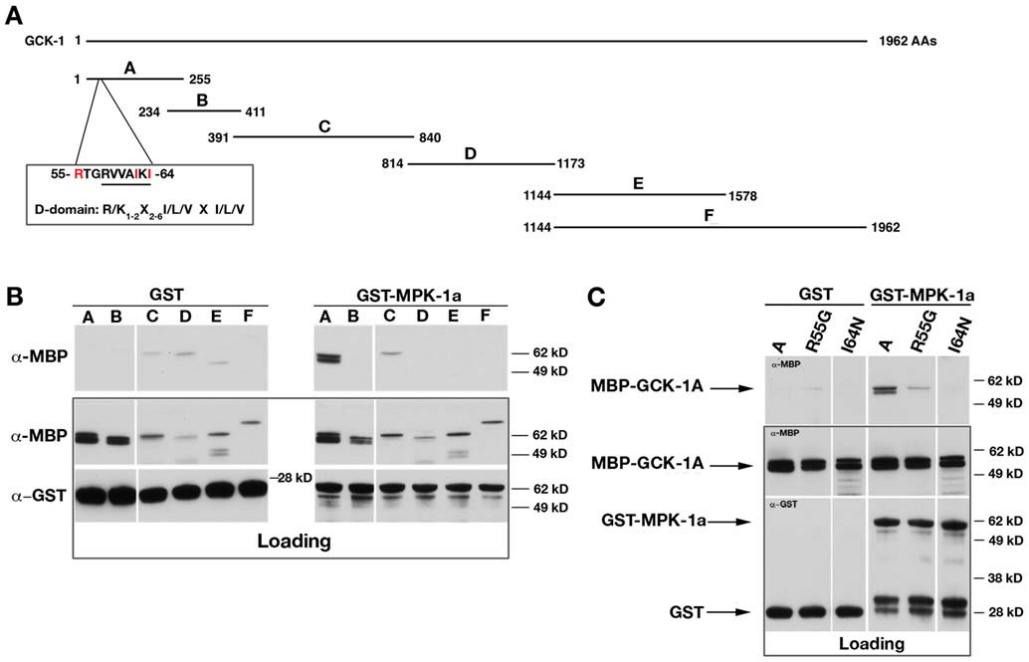
GCK-1 binds to MPK-1 *in vitro.* A) Schematic of the MBP-GCK-1 fragments used in the GST-MPK-1 binding assay (B). The putative ERK docking site in the GCK-1 N-terminus is shown. B) MBP-GCK-1 protein fragments (A) were incubated with GST or GST-MPK-1 coated glutathione beads. Top panel: α-MBP western analysis of washed beads. Lower panel: α-MBP and α-GST western analysis of protein loading in each binding reaction (0.5% of load). Molecular weights are indicated. C) The wt MBP-GCK-1A protein fragment and two different ERK docking motif mutant GCK-1A proteins were incubated with GST and GST-MPK-1 coated beads as in (B). Top panel: α-MBP western analysis of washed beads. Lower panel: α-MBP and α-GST western analysis of protein loading in each binding reaction (1.0% of load). Molecular weights are indicated.

The GCK-1 N-terminus harbors a predicted ERK docking site [46]. To determine whether MPK-1 binds directly to GCK-1 via this site, various GCK-1 protein fragments were expressed as maltose-binding protein (MBP) fusion proteins, purified from bacteria, and mixed with GST- or GST-MPK-1a-coated glutathione beads (Figure 6A,B). Of the six GCK-1 fragments tested, only the N-terminal fragment bound specifically to MPK-1a (Figure 6B). To determine whether the ERK docking site was necessary for this interaction, two separate docking site mutations were introduced into the GCK-1 N-terminal fragment (R55G and I64N). Both mutations resulted in a significant loss of GCK-1 binding (Figure 6C). These data indicate that GCK-1 and MPK-1 can directly interact through an ERK docking site in the GCK-1 N-terminus, suggesting that GCK-1-mediated inhibition of MPK-1a activation may occur via direct binding/sequestration of the MPK-1a isoform.

### GCK-1 loss results in increased germ cell death

**Figure 7:**
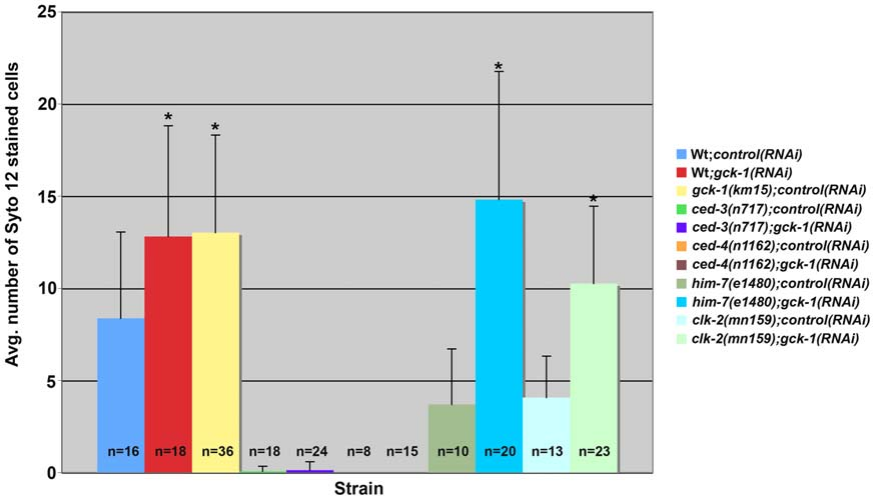
Germ cell death is increased in *gck-1(lf)* gonads. The average number of Syto12 stained apoptotic cells per hermaphrodite gonad arm of the indicated genotypes/conditions assayed 24 hours past the L4 stage. (*P<0.001: *(gck-1(RNAi)* and *gck-1(km15)* compared to Wt;*control(RNAi)*; *him-7(e1480);gck-1(RNAi)* and *clk-2(mn159);gck-1(RNAi)* compared to *him-7(e1480);control(RNAi)* and *clk-2(mn159);control(RNAi)* respectively. Mean ± standard error of the means; error bars represent the standard error of the means.

Under normal growth conditions, approximately 50% of developing *C. elegans* oocytes undergo physiological cell death [27]. This apoptosis requires progression through pachytene and MAP kinase activation [27]. Since GCK-1 is a negative regulator of MAP kinase signaling in the germline, we examined the frequency of germ cell apoptosis in wt and *gck-1(lf)* animals by staining live animals with Syto12, a dye that is specific for apoptotic cells [27]. At 24 hours past the L4 stage (L4+24 hours), *gck-1(RNAi)* and *gck-1(km15)* gonads had a statistically significant increase in apoptotic germ cells over wt controls (Figure 7). This germ cell death was dependent on the apoptotic machinery, as no dying cells were detected in *ced-3* or *ced-4* mutant animals (Figure 7)[27].

We reasoned that the increase in cell death in *gck-1(lf)* gonads could be due to an upregulation of the physiological germ cell death pathway or to introduction of cell damage that triggered a checkpoint-dependent apoptotic response. The *C. elegans* checkpoint genes *him-7* and *rad-5/clk-2* mediate apoptosis in response to DNA damage. Mutations in these genes decrease DNA damage-induced but not physiological germ cell death [47]. The frequency of germ cell death in *him-7(e1480)* and *clk-2(mn259)* hermaphrodites depleted of GCK-1 was significantly higher than control treated animals (Figure 7), indicating that the increase in germ cell death in *gck-1(lf)* animals is not mediated by the DNA checkpoint pathway. Altogether, these results suggest that GCK-1 is an inhibitor of physiological apoptosis in the *C. elegans* germline.

## Discussion

Here we demonstrate that the *C. elegans* germinal center kinase GCK-1 is required to inhibit abnormal oocyte development and germ cell apoptosis. Furthermore, loss of GCK-1 activity results in ectopic activation of a specific MPK-1 isoform that has not previously been shown to have a significant role in the germline. An ERK docking site dependent in vitro interaction between GCK-1 and MPK-1 suggests that GCK-1 may physically sequester MPK-1 from activation. These results are in striking contrast to the reported functions of other germinal center kinase family members, the majority of which have been shown to induce MAP kinase activation and apoptosis [2], [7], raising the question as to whether different germinal center kinases have disparate functions in different organisms or developmental contexts. This view is supported by the recent discovery that the Drosophila GCK-III kinase family member is a negative regulator of EGFR/ERK activation in fly neurons [48]. Loss of GCK-III/Happyhour leads to increased ERK activation and reduced sensitivity to alcohol sedation. Although the mechanism of Happyhour mediated inhibition of ERK activation is unknown, these findings are consistent with our data and reveal a conserved role for GCK-III family members in the inhibition of ERK signaling pathways.

MAP kinase activation is an essential component of oogenesis in many organisms [49]–[53]. Although strongly correlated with oocyte maturation in *C. elegans*, the requirement for MAP kinase activation in this process has been difficult to assess due to the essential role of MPK-1 in pachytene progression [24]–[26]. Moreover, the minor difference between MPK-1a and MPK-1b has made it difficult to study the specific function and localization of each isoform. Previous studies revealed that MPK-1b activation is specific to the germline. However, there was no discernable difference in MPK-1a activation in the presence or absence of germ cells, suggesting that MPK-1a functions may be restricted to the soma [44]. Whilst there is little doubt that MPK-1b plays a key role in the germline, we conclude that MPK-1a is also critical for germ cell development. While other models are formally possible, the simplest interpretation of our data is that the MPK-1a and MPK-1b isoforms are activated at distinct stages of oocyte development. MPK-1a is activated in mid-pachytene by an unknown signal, while MPK-1b is activated by MSP in maturing oocytes residing close to the spermatheca. We hypothesize that inappropriately high MPK-1a activation at mid-pachytene in the absence of GCK-1 leads to abnormal oocyte development and increased apoptosis.

Significantly fewer germ cells populate *gck-1(lf)* gonads as compared to wild type *C. elegans*. Interestingly, LET-60/Ras gf alleles or loss of a MAP kinase inhibitor, LIP-1, also result in fewer germ cells [43], [54]. Hence, the decreased germ cell population in *gck-1(lf)* gonads might also be attributed to inappropriately high MAP kinase activity. Conversely, GCK-1 could be affecting cell division and/or cell survival via a MAP kinase independent pathway. Indeed, other germinal center kinases, including Drosophila Slik and Hippo, play critical roles in cell cycle progression and growth control through novel signaling pathways [19], [55]. Interestingly, both the Slik and Hippo kinases are regulated in part by Raf, although independently of Raf's role as an activator of the MAP kinase pathway [16], [19], [56]. However, unlike GCK-1, both of these kinases act to limit cell proliferation, and loss-of-function mutants display gross tissue overgrowth [19], [56].

Deciphering the exact mechanism through which GCK-1 affects germ cell development and apoptosis will require the identification of GCK-1 binding partners and the physiological substrates of this interesting kinase. This study contributes to a growing body of evidence suggesting that the in vivo functions of the Germinal Center kinases are likely to vary in different developmental and cellular contexts. Hence the analysis of each member of this large kinase family in a variety of model organisms will be necessary to elucidate these functions and their contribution to human development and disease.

## Materials and Methods

### cDNAs

The *C. elegans* genome database predicts four alternatively spliced *gck-1* isoforms (http://wormbase.org Release WS203, June 29, 2009). The *gck-1a* isoform was PCR amplified from a *C. elegans* cDNA library and DNA sequencing confirmed that it was identical to the predicted *gck-1a* cDNA (Genbank: AAC69038.1). All of the relevant experiments described here were performed with this cDNA.

### Nematode strains and culture methods

Standard techniques were used to culture and genetically manipulate all strains [57]. All mutant strains are derivatives of the wt strain *C. elegans* var. Bristol. The following strains were maintained at 20°C: N2 (wild-type), NL2098 (*rrf-1(pk1417)*I), MT1522 (*ced-3(n717)*IV), MT2547 (*ced-4(n1162)*III), CB1480 (*him-7(e1480)*V), SP506 (*rad-5/clk-2(mn159)*III), CB4801 (*fog-2(q71)*V), JS347 (*gck-1(km15)*/mIs10V) (mIs10: myo-2::GFP expressing Chr. V balancer that results in GFP positive muscle cells [58]), JS345 (*gck-1(km15)*V*/*nT1[qIs51](IV;V)). nT1[qIs51] was used as a dominant green fluorescent balancer chromosome, allowing *qIs51*-containing animals to be scored beginning at the four-cell stage of embryogenesis [59], [60]. SS104 (*glp-4(bn2ts)*I) is germline-less at 25°C and was therefore maintained at 15°C. The *gck-1(km15)* deletion mutant was generated by a TMP/UV method and isolated using a sib-selection protocol [61]. The sequences flanking the breakpoint are: GAATACTTTTGTG and TATTACACTTTT, corresponding to a 1334 base pair deletion between nucleotides 13646 and 14969 in cosmid T19A5. Direct sequencing of the PCR products was used to verify the deleted region.

### RNA Mediated Interference (RNAi)

To create the *gck-1a* and *mpk-1(RNAi)* constructs, their entire coding sequences were PCR-amplified from a *C. elegans* cDNA library using primers corresponding to their start and stop codons with restriction sites added for subcloning. *gck-1a* primers: 5′-CGTATCTAGAATGACAACGACATCATCAG-3′, and 5′-GCATAAGCTTTTACCGGGGTTGTCAGTC-3′. *mpk-1* primers: 5′-GTACGTCGACGCATGCCAACGTGGATACC-3′, and 5′-CTGAGCGGCCGCCTAAACAGGATTCTGCCTC-3′. For *lin-45* and *ksr-2*, corresponding EST cDNA clones (yk357d2 and yk343d6 respectively (gifts from Y. Kohora, National Institute of Genetics, Japan)) were PCR amplified using primers with restriction sites for subcloning. All resulting cDNAs were confirmed by sequencing, subcloned into the L4440 feeding plasmid, and transformed into HT115 (DE3) *E. coli* [62], [63]. The *mek-2*/L4440 construct was obtained from the *C. elegans* feeding library (Geneservice/Source Bioscience, Nottingham, UK). dsRNA production was induced by isopropyl-beta-D-thiogalactopyranoside (IPTG) and fed to *C. elegans* hermaphrodites as previously described [63]. Control RNAi was the L4440 feeding plasmid without an insert. Microinjection of dsRNA corresponding to full-length *gck-1a* resulted in a phenotype that was indistinguishable from *gck-1(RNAi)* by feeding.

All primers were synthesized by SigmaGenosys (Woodlands, TX). All sequencing was performed by the M.D. Anderson DNA Analysis Core Facility (Houston, TX), which is supported by NCI Grant CA-16672.

### Microscopy

A Leica DMR compound fluorescent microscope (Leica Microsystems, Wetzlar, Germany) equipped with a Spot digital camera (Diagnostic Instruments, Sterling Heights, MI) was used to capture Differential Interference Contrast (DIC) and immunofluorescent images. For DIC microscopy, animals were anesthetized in 1x anesthetic (0.1% tricaine, 0.01% tetraimidizole, 0.1% Tween in M9), dissected on a Poly-L-Lysine coated glass slide and covered by a 22×22 mm glass coverslip mounted on petroleum jelly.

Immunofluorescent image stacks (0.2 uM per slice) were obtained using a 40X/1.3 or 60X/1.45 oil immersion lens attached to a Nikon 2000U inverted microscope equipped with a motorized x, y, and z-stage (Nikon, Melville, NY), and a CoolSnap HQ camera (Roper Scientific, Tucson, AZ). Images were captured with Metamorph software (Molecular Devices, Sunnyvale, CA) and deconvolved using AutoDeblur (AutoQuant, Troy, NY). Confocal image-stacks (12 slices per gonad) were obtained using a Zeiss LSM 510 confocal microscope with a 63X/1.4 oil immersion lens. Adobe Photoshop CS2 (Adobe Systems Inc., San Jose, CA) was used to prepare all figures. Within each figure, the exposure time and any post-microscopy processing (AutoDeBlur, Metamorph, and Photoshop) were identical for each gonad.

### Antibodies and Immunofluorescence

The anti-phospho-histone H3 (pH 3; Upstate Biotechnology, Waltham, MA) [40], anti-RNAP-II CTD phospho-serine 5 (H14; Covance, Denver, PA) [37], and anti-diphosphorylated ERK-1&2 (MAPK-YT; Sigma, St. Louis, MO) [25], have been previously described. Goat anti-rabbit and anti-mouse IgG (H+L) conjugated to Rhodamine Red-X and Alexa Fluor 488 anti-mouse secondary antibodies were obtained from Invitrogen (Carlsbad, CA). Fluorescein goat anti-rabbit IgG (H+L) and IgM (mu chain specific) secondary antibodies were obtained from Vector Laboratories (Burlingame, CA).

For immunofluorescence analysis of *C. elegans* germlines, adult (L4+24 hours) animals were transferred to a watch glass containing 1x anesthetic. Gonads were extruded using two 22½G-syringe needles by slicing the animals just below the pharynx. Dissected animals were transferred by mouth pipetting in 1x PBS +0.1% Tween to a glass culture tube where they were washed, and subsequently fixed in 3% formaldehyde and 0.1 M potassium phosphate for one hour, followed by post-fixation in −20°C methanol for 5 minutes as previously described [43]. After washing, specimens were incubated with the appropriate primary antibody overnight at room temperature (RT). The following day, the gonads were washed and incubated with secondary antibodies for one hour at RT. Animals were then mounted in Vectashield mounting media containing 4′,6-Diamidino-2-phenylindole (DAPI; Vector Laboratories) on a microscope slide and gently covered with a coverslip.

For MAPK-YT immunostaining, dissections were performed as above, while fixation and MAPK-YT staining was performed as previously described [64], with the exception that dissected gonads were immediately fixed in 4% paraformaldehyde and 0.1 M potassium phosphate overnight at 4°C. The following day, gonads were washed, incubated with primary antibody for 4 hours at RT, washed again, and incubated with secondary antibody overnight at 4°C as previously described [64]. Gonads were then treated with RNAse, stained with To-Pro-3 (Invitrogen), washed in water, and mounted on a microscope slide in Slow Fade Gold mounting media (Invitrogen). Confocal images were captured and MAPK-YT immunostaining intensity was quantified using Imaris X64 5.0.1 software (Bitplane Inc, St. Paul, MN) as follows: an isosurface of pachytene MAPK-YT immunostaining was created for each image (threshold = 29) and the mean intensity for each gonad recorded and plotted.

### Nuclei Counts

Animals were dissected at L4+24 hours, fixed in 3% paraformaldehyde and 0.1 M potassium phosphate for one hour, and stained with DAPI. Image stacks were acquired and deconvolved as described above. Nuclei were counted using the manual count option in Metamorph. Nuclei counts for each region were compared to wt using Dunnett's Statistical Test using a 99% family confidence interval [65].

In wt gonads, condensed nuclei in the distal region that had a metaphase chromosome arrangement were counted as mitotic metaphase nuclei. The mitotic region as a whole was defined as nuclei residing from the distal tip up to the transition zone [66]. The transition zone was defined as the region containing crescent-shaped nuclei [33], [67]. Pachytene nuclei were counted based on chromosome morphology [68]. Nuclei spanning from the end of the pachytene region to the spermatheca were counted as diplotene and diakinetic nuclei.

The *gck-1* loss-of-function (lf) germline nuclei were counted using the same methods, except that nuclear morphology was the only criteria used for distinguishing different stages since the temporal and spatial order of developing germ cell nuclei was severely disrupted from pachytene to diakinesis in *gck-1(lf)* gonads. Some nuclei did not have distinct chromosome morphology and were therefore grouped as “other.”

### Western Analysis

100 adult (L4+24 hours) animals were individually picked into M9 solution in a microfuge tube, allowed to purge for three to five hours, and washed three times in M9. Animals were then resuspended in SDS-PAGE loading buffer [69], boiled for five minutes, and centrifuged before loading the supernatant on an SDS/PAGE gel. One tube of 100 worms was used per lane. Separated proteins were transferred to an Immobilon-NC membrane (Millipore, Billerica, MA), and blocked in 1x western wash buffer (20 mM Tris, pH 8.0, 150 mM NaCl, 0.1% Tween-20, and 0.01% SDS) plus 5% nonfat dry milk. Antibodies (anti-diphosphorylated ERK-1&2) (MAPK-YT) and anti-α-tubulin (Sigma, St. Louis, MO) were diluted in 1x western wash buffer plus 1% milk. After washing, bound antibodies were visualized using chemiluminescence (ECL; GE LifeSciences, Piscataway, NJ).

### In vitro binding assays

DNA sequences corresponding to the GCK-1 protein fragments in Figure 6A were PCR amplified from a full-length GCK-1 cDNA and subcloned into a pMAL-Maltose Binding Protein (MBP) *E. coli* expression vector (New England Biolabs (NEB), Beverly, MA). The MBP-GCK-1 fusion proteins were expressed and purified on amylose beads following manufacturer's instructions (NEB). A Glutathione-S-Transferase (GST)-MPK-1a fusion protein was created by PCR amplification of the MPK-1a open-reading frame from a *C. elegans* cDNA library. The MPK-1a cDNA was subcloned into the pGEX-6P-1 *E. coli* expression vector (GE LifeSciences). GST and GST-MPK-1a proteins were expressed and purified from *E. coli* using glutathione beads (GE LifeSciences) following manufacturer's instructions, except that the proteins were not eluted from the beads. The GST and GST-MPK-1a coated beads were incubated with approximately 2 ug eluted MBP-GCK-1 fusion protein in 100 ul binding buffer (200 mM Tris pH 8.0, 200 mM NaCl, 1 mM EDTA and 0.5% Triton X-100, 100 ug/ml BSA) for more than 2 hours at 4°C. The beads were washed four times in binding buffer, resuspended and boiled in SDS/PAGE buffer, and the bound proteins separated on a 10% Tris-glycine polyacrylamide gel. A fraction of the protein in each binding reaction was also loaded on the gel. Separated proteins were transferred to nitrocellulose and subjected to western analysis with α-MBP (NEB) and α-GST [70] antibodies. To create the ERK docking site mutations, the MBP-GCK-1A construct was subjected to PCR-based site-directed mutagenesis (Stratagene/Agilent Technologies, Santa Clara, CA). Binding reactions with these proteins were carried out as described above.

### Cell Death Assays

Cell death assays were performed by staining hermaphrodite animals (L4+24 hours) with Syto12 (Invitrogen) as previously described [27]. Data were analyzed using both the Kruskal-Wallis Test and Two-Way Analysis of Variance (ANOVA) at a 99% confidence level. Results from both statistical tests were consistent.

## Supporting Information

Table S1 Germ nuclei counts
(0.03 MB DOC)

Figure S1 An alignment of GCK-1 and other GCK-III subfamily protein sequences. The GCK-III subfamily was defined in (Dan et al., 2001). The aligned sequences are human MASK (GenBank: BAA92785.2); human MST3 (Swiss-Prot: Q9Y6E0.1); human SOK1/YSK1 (Swiss-Prot: O00506.1); Drosophila GCKIII (GenBank: AAF55388.1); and C. elegans GCK-1a (GenBank: AAC69038.1). The sequences were aligned using the ClustalW2 EMBL-EBI server: http://www.ebi.ac.uk/Tools/clustalw2/index.html) and BOXSHADE 3.21 (http://www.ch.embnet.org/software/BOX_form.html). Identical amino acids are in solid boxes and similar residues are shaded. The kinase domain is underlined and the GCK-III subfamily signature sequence is overlined. The breakpoints in the gck-1(km15) allele are indicated (*).
(1.03 MB TIF)

Figure S2 Schematic of the C. elegans gonad. The C. elegans hermaphrodite gonad consists of two mirror image U-shaped arms. Each arm consists of a distal mitotic region (*) with proliferating germ cells. These nuclei transition into meiosis with a crescent shape characteristic of leptotene and zygotene. Pachytene nuclei are arranged on the surface of the gonad and surround a common anucleate cytoplasm, the rachis. Germ cells remain in the pachytene stage for an extended period before passing through diplotene (condensing chromosomes enclosed in a nuclear membrane) and arresting in diakinesis (six bivalent chromosomes in a nuclear membrane). In response to MSP, the oocyte most proximal to the spermatheca (S) matures: the nucleus migrates distally and nuclear envelope breakdown occurs. The mature oocyte is then ovulated through the spermatheca (S) where it is fertilized and passed into the uterus where the embryo (E) develops before being extruded into the environment.
(9.19 MB TIF)

Figure S3 Germ cell numbers are reduced in gck-1(lf) hermaphrodites. (A) The average total number of germline nuclei in a single gonad of the indicated genotype as grouped by nuclear stage (see Materials and methods). (B) The average number of mitotic metaphase nuclei per gonad arm. (n = 4 for each genotype; **P<0.001; error bars represent standard error of the means.)
(0.74 MB TIF)

Figure S4 The gck-1(lf) phenotype requires the MAP kinase pathway. (A–H) DAPI stained gonads from (A,C,E,G) wt and (B,D,F,H) gck-1(km15) animals fed control (A,B), ksr-2 (C,D), mek-2 (E,F), or lin-45 (G,H) dsRNA. Scale Bar, 20 Î¼m.
(1.61 MB TIF)
