# Supplementary material for: The Germinal Center Kinase GCK-1 Is a Negative Regulator of MAP Kinase Activation and Apoptosis in the C. elegans Germline
Source: PLoS One. 2009 Oct 14;4(10):e7450. doi: 10.1371/journal.pone.0007450 (PMC2757678; doi:10.1371/journal.pone.0007450)
Supplement: Table S1 — Germ nuclei counts (0.03 MB DOC) [file pone.0007450.s001.doc]

Supplemental Table S1: Germ nuclei counts

|  | Genotype/Condition | | | |
| --- | --- | --- | --- | --- |
| Region | Wt | *gck-1(km15)*/nT1 | *gck-1(RNAi)* | *gck-1(km15)* |
| mitotic metaphase | 5.8±1.4 | 4.5±1.3 | 4.5±0.3 | 2.8±1.0 |
| distal mitotic region | 183. 0±9.6 | 162.8±14.6 | **105.8±6.2 | **11.5±5.8 |
| transition zone | 118.0±5.4 | 101.5±6.2 | **58.8±1.5 | **45.3±16.2 |
| pachytene region | 709.0±30.9 | **462.5±34.6 | **287.3±23.5 | **132.3±20.8 |
| diplotene and diakinesis | 25.8±4.0 | 26.4±3.2 | 17.3±3.5 | **5.8±1.3 |
| unidentified | 0.0 ± 0.0 | 0.0±0.0 | **47.0±9.2 | **84.3±14.9 |
| **Total** | **1041. 0±36.4** | ****757.5±43.0** | ****520. 0±27.2** | ****351.5±24.5** |

n=4 gonad arms for each genotype; numbers are given as mean ± standard error of the means.

** Significantly different from wild type at a 99% confidence interval; P<0.001
