# Supplementary figures and images for: The Germinal Center Kinase GCK-1 Is a Negative Regulator of MAP Kinase Activation and Apoptosis in the C. elegans Germline
Source: PLoS One. 2009 Oct 14;4(10):e7450. doi: 10.1371/journal.pone.0007450 (PMC2757678; doi:10.1371/journal.pone.0007450)

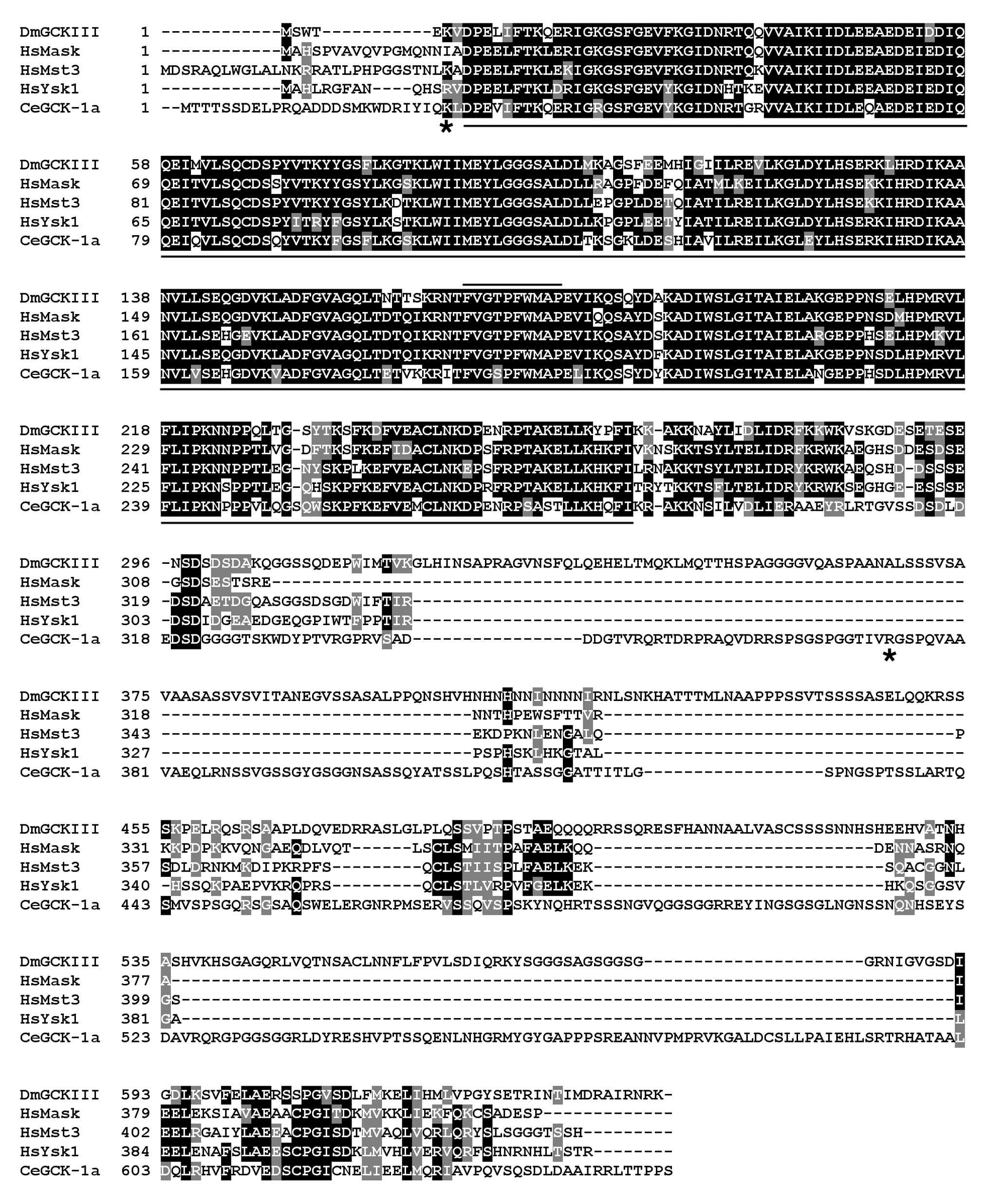

Supplement: Figure S1 — An alignment of GCK-1 and other GCK-III subfamily protein sequences. The GCK-III subfamily was defined in (Dan et al., 2001). The aligned sequences are human MASK (GenBank: BAA92785.2); human MST3 (Swiss-Prot: Q9Y6E0.1); human SOK1/YSK1 (Swiss-Prot: O00506.1); Drosophila GCKIII (GenBank: AAF55388.1); and C. elegans GCK-1a (GenBank: AAC69038.1). The sequences were aligned using the ClustalW2 EMBL-EBI server: http://www.ebi.ac.uk/Tools/clustalw2/index.html) and BOXSHADE 3.21 (http://www.ch.embnet.org/software/BOX_form.html). Identical amino acids are in solid boxes and similar residues are shaded. The kinase domain is underlined and the GCK-III subfamily signature sequence is overlined. The breakpoints in the gck-1(km15) allele are indicated (*). (1.03 MB TIF) [file pone.0007450.s002.tif]

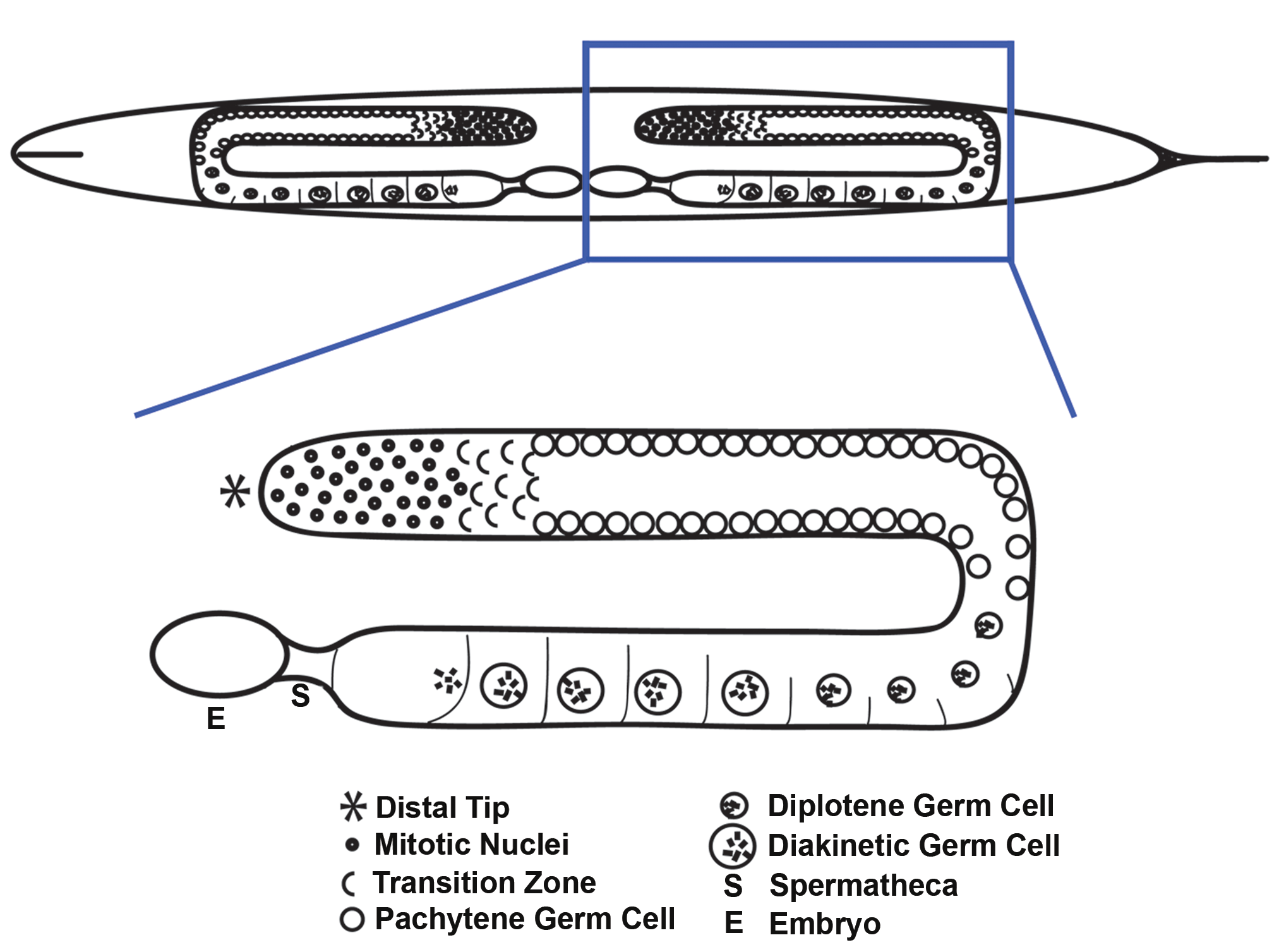

Supplement: Figure S2 — Schematic of the C. elegans gonad. The C. elegans hermaphrodite gonad consists of two mirror image U-shaped arms. Each arm consists of a distal mitotic region (*) with proliferating germ cells. These nuclei transition into meiosis with a crescent shape characteristic of leptotene and zygotene. Pachytene nuclei are arranged on the surface of the gonad and surround a common anucleate cytoplasm, the rachis. Germ cells remain in the pachytene stage for an extended period before passing through diplotene (condensing chromosomes enclosed in a nuclear membrane) and arresting in diakinesis (six bivalent chromosomes in a nuclear membrane). In response to MSP, the oocyte most proximal to the spermatheca (S) matures: the nucleus migrates distally and nuclear envelope breakdown occurs. The mature oocyte is then ovulated through the spermatheca (S) where it is fertilized and passed into the uterus where the embryo (E) develops before being extruded into the environment. (9.19 MB TIF) [file pone.0007450.s003.tif]

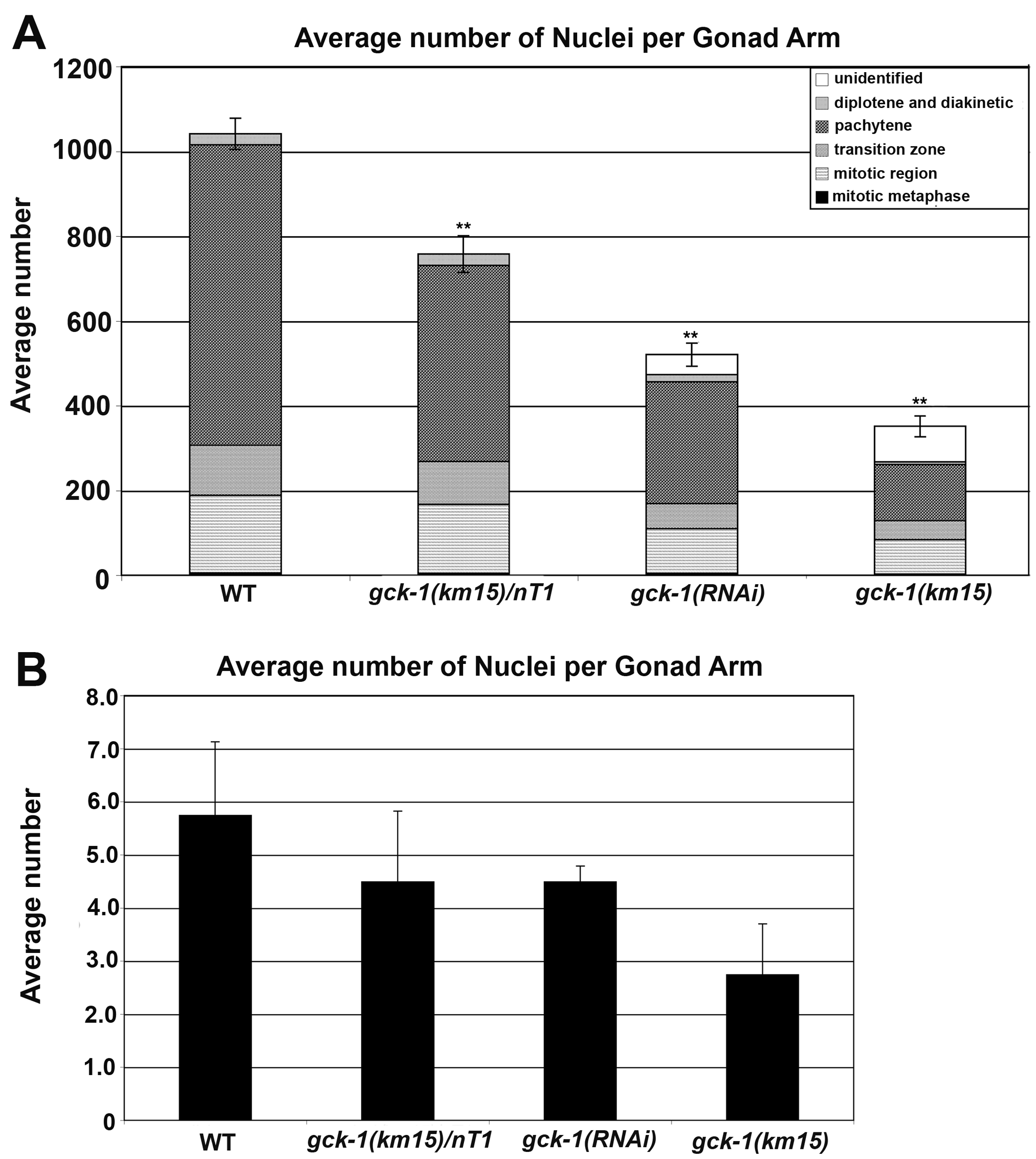

Supplement: Figure S3 — Germ cell numbers are reduced in gck-1(lf) hermaphrodites. (A) The average total number of germline nuclei in a single gonad of the indicated genotype as grouped by nuclear stage (see Materials and methods). (B) The average number of mitotic metaphase nuclei per gonad arm. (n = 4 for each genotype; **P<0.001; error bars represent standard error of the means.) (0.74 MB TIF) [file pone.0007450.s004.tif]

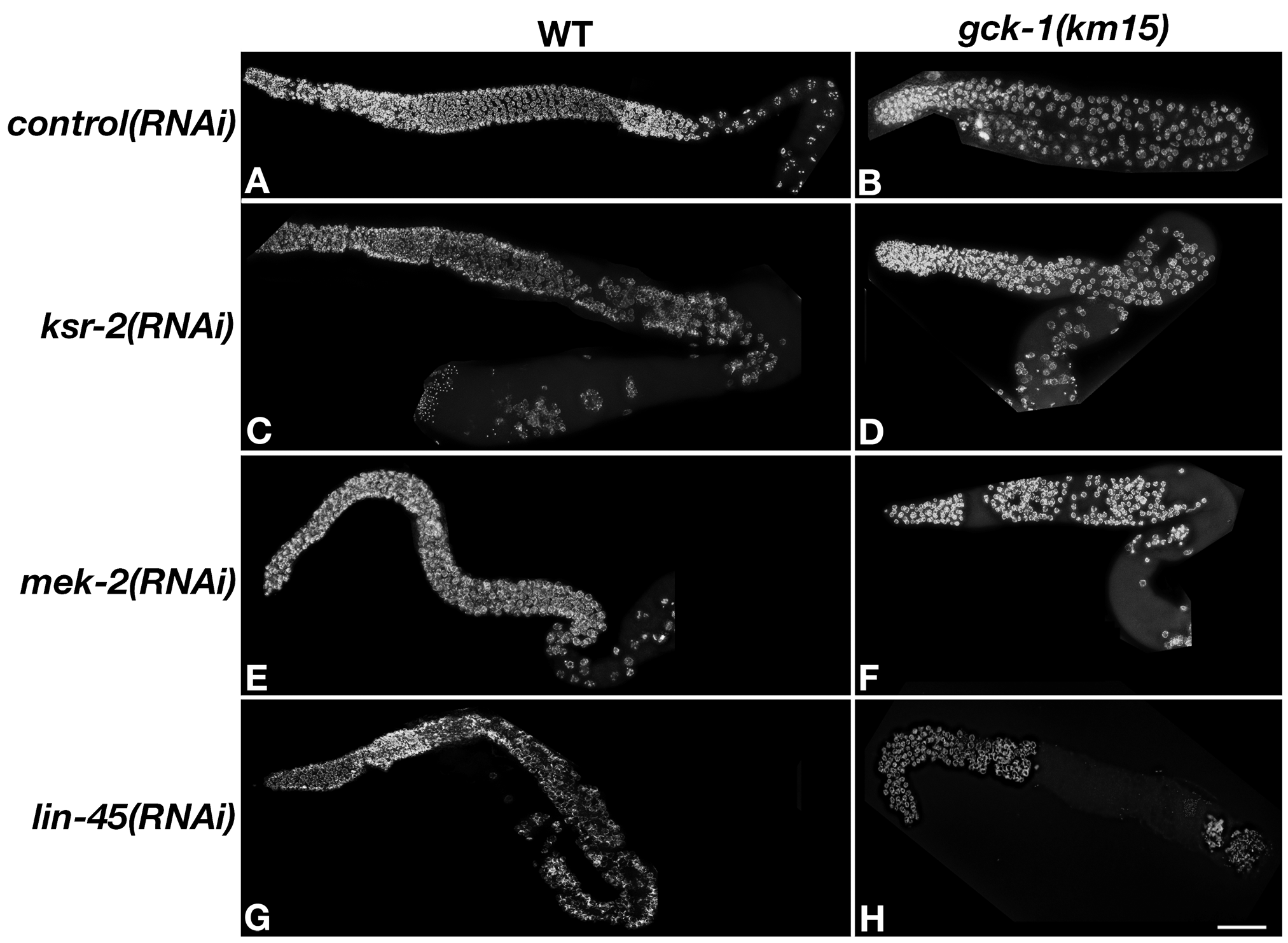

Supplement: Figure S4 — The gck-1(lf) phenotype requires the MAP kinase pathway. (A–H) DAPI stained gonads from (A,C,E,G) wt and (B,D,F,H) gck-1(km15) animals fed control (A,B), ksr-2 (C,D), mek-2 (E,F), or lin-45 (G,H) dsRNA. Scale Bar, 20 Î¼m. (1.61 MB TIF) [file pone.0007450.s005.tif]
